# Hip dips technique: Filling lateral depressions with hyaluronic acid of large particles

**DOI:** 10.1002/ski2.461

**Published:** 2024-09-26

**Authors:** Luciana Lourenco, Helena Medeiros, Luísa Ferreira, Natasha Favoretto Dias de Oliveira, Roberta Lopes, Rosa Sigrist

**Affiliations:** ^1^ Private Practice in Dermatology Sao Paulo Brazil; ^2^ Faculdade de Ciencias Medicas de Minas Gerais Belo Horizonte Minas Gerais Brazil; ^3^ Universidade de Sao Paulo Hospital das Clínicas da Faculdade de Medicina da USP São Paulo Brazil

## Abstract

**Introduction:**

Hip dips, often referred to as the pronounced trochanteric depression, can be caused by athletic muscular definition or the ageing process. This depression might impact the desirable contour of the buttocks in some female patients.

**Patients and Methods:**

A technique is described for female patients exhibiting moderate to severe degrees of lateral trochanteric depression. This technique utilizes a specific marking, a well‐thought‐out plan and a designated product. Eleven patients were selected to use this technique to improve their trochanteric depression and enhance their buttocks contour without undergoing surgery. The product used was Sofiderm Subskin, which was applied at the intermediate subcutaneous layer.

**Results:**

The authors report favourable aesthetic results with the proposed technique, and the patients expressed high satisfaction levels.

**Discussion:**

Though safe for injection, the lateral gluteal depression can still be challenging to expand. The product chosen has both a high G prime and a large molecular size, contributing to its resistance to deformation.

**Conclusion:**

The hip dips technique using hyaluronic acid for the augmentation of lateral depression has shown to be minimally invasive; it provides quick results without significant risks or downtime.

## Graphical abstract

Description of the hip dips technique using hyaluronic acid for augmentation of the lateral depression. The minimally invasive technique proved to be safe and presented rapid results without major risks and downtime. The technique is characterized with a special marking, a safe plan, and a specific product (large size particles). The authors used a special ultrasound to choose the best plan for application. The patients were followed up for 10 months and presented no complications.

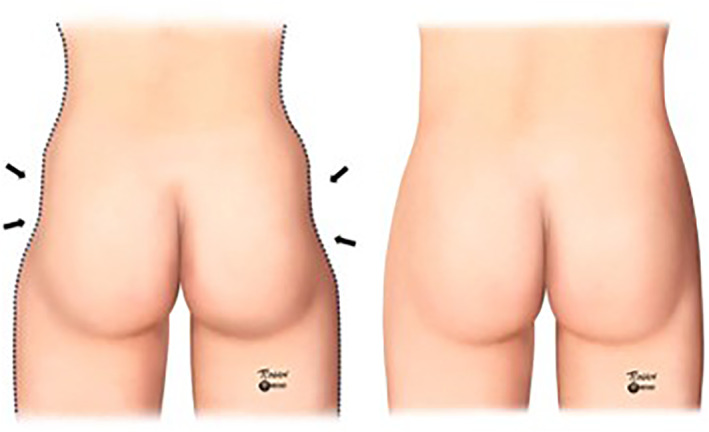

**What is already known?**

- The hips dips depression might compromise the desirable buttocks contour in some female patients.
- The buttock area has received media attention in recent years, increasing patient demand for buttock reshaping or contouring and augmentation.
- Hyaluronic acid (HA) has been used to enhance the buttocks. It is able to promote pro‐collagen, stimulate growth factors in the skin and induce mesenchymal stem cell migration in the extracellular matrix.

**What does this study add?**

- A new and innovated technique: the hip dips technique using HA for augmentation of the lateral depression.
- The technique uses a new special marking, with a safe plan and a special ultrasound to choose the best plan for application.
- The technique uses a specific product: HA with large size particles.

## 1 INTRODUCTION

The buttock area has garnered media attention in recent years, leading to an increased patient interest in buttock reshaping or contouring and augmentation procedures. As a result, a structured assessment of individual gluteal anatomy and the development of non‐surgical methods to enhance aesthetic appeal are essential. Certain well‐regarded characteristics of the female buttock include a smooth, rounded gluteal projection, a short intergluteal fold and an infra‐gluteal fold that extends to the mid‐thigh line. The waist‐hip ratio is typically considered attractive when it measures around 0.7. Furthermore, the lateral thighs should harmoniously extend the shape of the buttock and flanks and transition smoothly towards the buttocks, without irregularities or excess fat.^1^, ^2^

**FIGURE 1:**
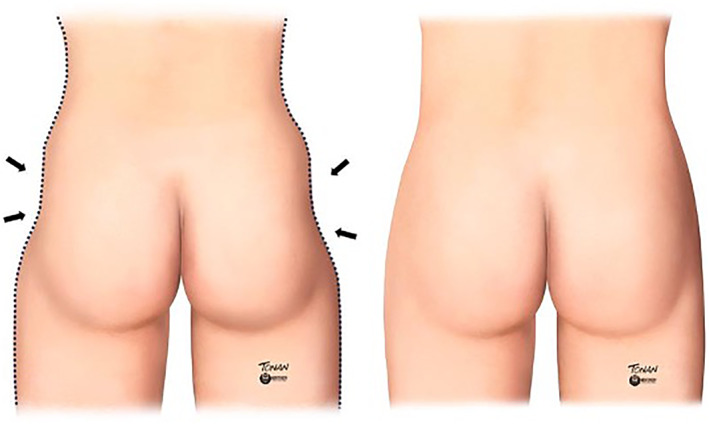
Pronounced trochanteric depression, popularly called ‘hip dips’.

Therefore, a noticeable trochanteric depression, commonly referred to as ‘hip dips’, may compromise the desirable buttock contour in some female patients (Figure 1). This depression could be constitutional, resulting from a pronounced muscular definition in athletes, or happen as part of the ageing process due to skin laxity and fat accumulation in the lower third of the buttocks. In men, this depression is an inherent characteristic of the android gluteal form.^1^, ^2^, ^3^

New aesthetic treatments are emerging to improve body contour. Mastery of gluteal anatomy is mandatory for safely performing gluteal augmentation procedures.

The trochanteric depression, found below the greater trochanter in the lateral buttock region, is composed of the greater trochanter and insertions of multiple thigh and buttock muscles. These include the gluteus medius, vastus lateralis, quadratus femoris and gluteus maximus.^3^

The safest place to inject HA in the gluteal region is the subcutaneous fat. This layer is devoid of blood vessels exceeding 2 mm in calibre or other significant structures susceptible to complications.^4^, ^5^, ^6^, ^7^ Larger calibre nerves and blood vessels are found in the muscular region or deeper, increasing the risk of procedures in this area.^3^, ^7^

Buttock contour surgery was first described by Pitanguy in 1964 to improve trochanteric lipodystrophy. Since then, procedures such as gluteal implants, autologous fat grafting and flap surgery have been performed.^1^, ^8^, ^9^

Apart from surgery, there are other minimally invasive gluteal treatments, such as collagen biostimulators. Poly‐L‐lactic acid is also used to create volume by increasing dermal thickness.^10^, ^11^

Recently, HA has been employed to enhance buttocks. The first HA used for buttock enhancement was Macrolane in 2013.^9^, ^12^, ^13^, ^14^ The ideal HA for gluteal volumizing should possess specific rheological properties, including a high‐cohesion gel with the capacity to lift and provide smoothness. Furthermore, HA should yield both immediate filling and long‐term results.^15^, ^16^, ^17^ Previous studies also indicate that HA can stimulate pro‐collagen and growth factor production in the skin, as well as induce mesenchymal stem cell migration in the extracellular matrix.^18^, ^19^

## 2 PATIENTS AND METHODS

The authors described a technique intended for young female patients suffering from moderate to severe degrees of lateral trochanteric depression. This procedure involves a unique marking system, a secure plan and a specialized product. The selected 11 patients for this technique were females desiring to enhance trochanteric depression and improve buttock contour but were unwilling to undergo surgery.

The exclusion criteria were as follows: obesity, excessive muscle and skin laxity, local infections, previous hip prosthesis, allergy to HA or any known contraindications for fillings with HA.

The patients were evaluated both photographically and through ultrasound. They also completed a questionnaire regarding their level of satisfaction and any adverse effects experienced. Patients were followed up for an average duration of 10 months.

The product selected for this technique is Sofiderm Subskin. It possesses stable and large HA gel particles (800–1800 nm), with a high G prime (550 pa), available in 10 and 20 mL syringes. The authors only recommend using this product to treat the body.

The chosen plan involved the intermediate subcutaneous layer, and the application was guided by a high‐resolution ultrasound LOGIQ E10 (GE Healthcare) with a linear probe ranging from 6 to 24 MHz. This plan is safe, void of large vessels, or critical structures, contrary to the muscular and submuscular region, which houses larger calibre nerves and vessels, such as the superior and inferior gluteal veins.

**FIGURE 2:**
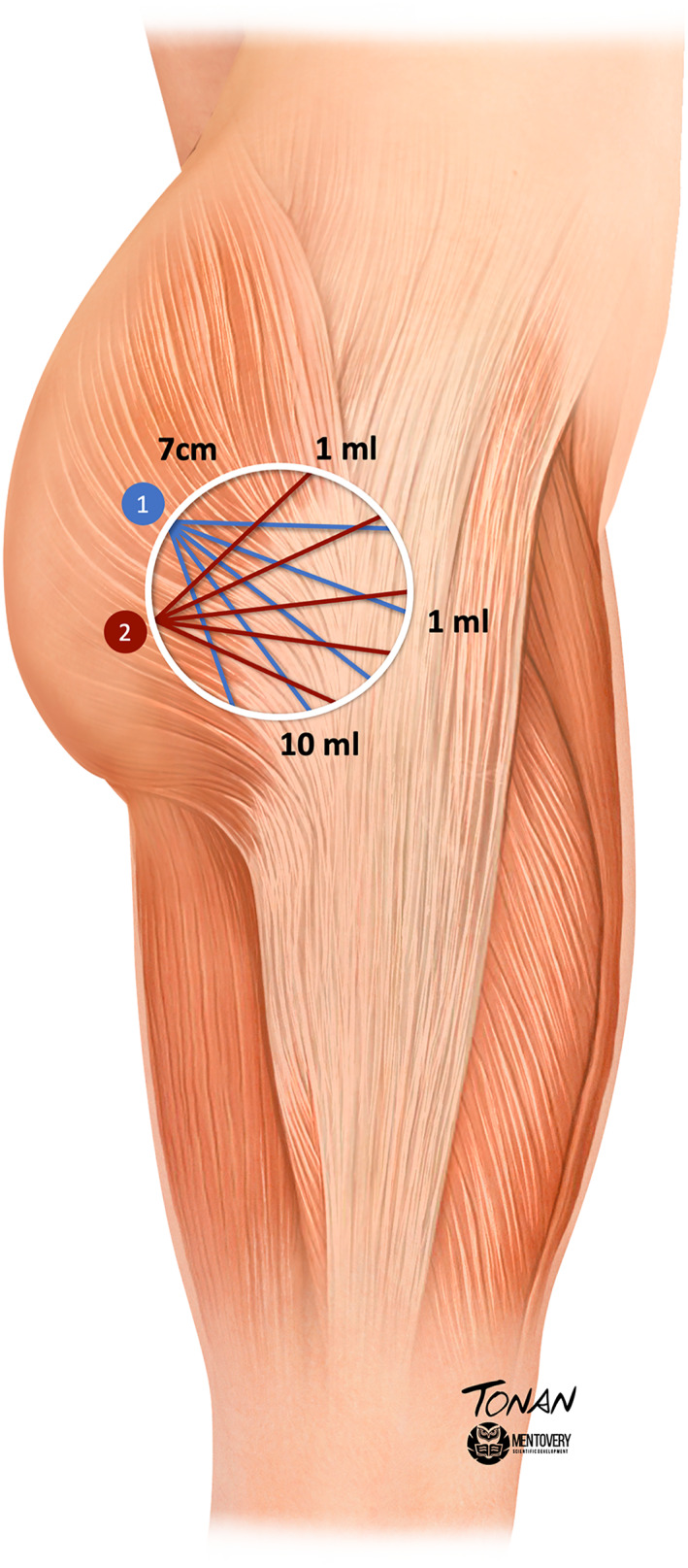
Hip dips technique representation: In the figure above, the circle represents the treated hip dips area, up to 7 cm in diameter. The numbers 1 and 2 represent the cannula inlet holes. From each point (1 and 2), five arms were designed to apply 1 mL of hyaluronic acid per fan arm, totalling 10 mL per side.

The authors propose the use of the ‘fan technique’, which involves two fans originating from the central medial region of the buttock. Using this method, they successfully covered a depression region up to 7 cm. The authors structured each fan with five arms, with each arm receiving 1 mL of HA via retro‐injection. This technique results in a total of 5 mL applied per fan and 10 mL per side (Figure 2).

Lidocaine‐filled anaesthetic buttons must be prepared to create the inlet holes. The application should be carried out using 3 mL syringes, which are attached to 18G × 70 mm cannulas.

After marking the area to be treated, the patient should be positioned in the lateral decubitus posture with flexed legs, and support should be placed between the legs to align the treatment area.

Prophylactic antibiotics were administered due to the use of an 18G × 70 cannula and the potential for subclinical folliculitis in the area. Patients were given a 1 g dose of azithromycin on the day of the procedure and were advised to abstain from sports for 2–3 days. The treatment can be repeated in 15 days if necessary.

The treated patients were asked to complete a questionnaire regarding their level of satisfaction and any adverse effects. Patients were followed up for an average duration of 10 months.

## 3 RESULTS

**FIGURE 3:**
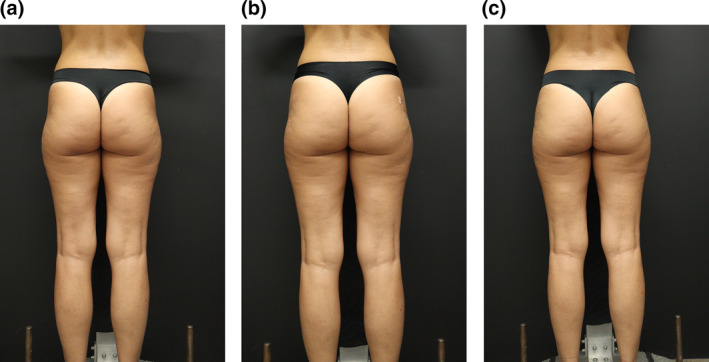
(a) Before treatment with evident bilateral trochanteric depression. (b) After the first session with 10 mL of HA filler on each side. (c) After 15 days, a second session was performed with 10 mL of HA filler on each side. HA, hyaluronic acid.

The authors observed favourable aesthetic results with the proposed technique (Figures 1 and 2). Mild bruising and pain were noted at the procedure site for 2–3 days post‐operation. No serious adverse events were reported, with the majority of treatment‐related adverse events being mild to moderate and transient. Figure 3 illustrates a reduction in the lateral trochanteric depression, along with an enhancement in the contour of the buttocks after two sessions, spaced 15 days apart. Patients declared high levels of satisfaction, noticing an improvement in their quality of life.

**FIGURE 4:**
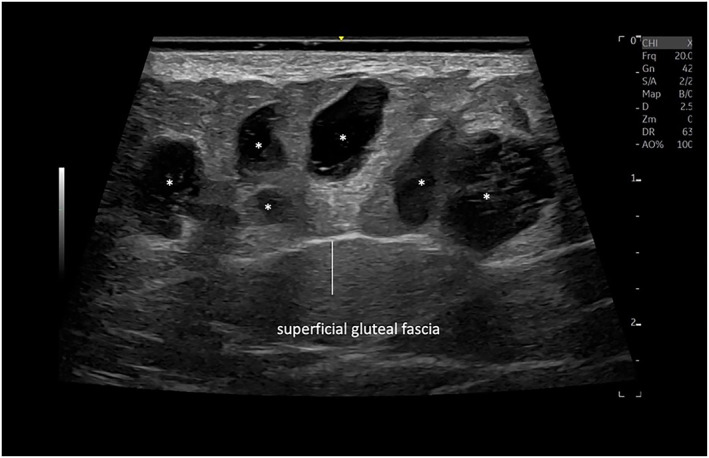
Sonographic image of the hip dips using a multifrequency linear probe ranging from 6 to 24 MHz demonstrates anechoic pseudo cystic deposits (*) above the superficial gluteal fascia of the hip dips.

Sonographic images were taken to demonstrate that the product was injected above the superficial gluteal fascia (Figure 4).

## 4 DISCUSSION

The contour of the gluteal region is relevant to attractiveness.^5^ Prior studies have shown that enhancements in body contour directly and positively affect a patient's quality of life.^20^, ^21^

Disharmonious lateral thighs and an abrupt curvature between the flanks and buttocks, which form a depression, are considered unaesthetic—this is particularly true among Hispanic and African American ethnicities.^1^, ^2^

The authors treated only female patients because the shape of the gluteal region in men is different, appearing square while standing and butterfly‐shaped during contraction, unlike the rounded shape seen in females. The male trochanteric depression, situated below and lateral to the gluteus medius, is a crucial area responsible for the final masculine appearance. This area where fat does not accumulate is crucial to be well‐defined, as it delineates the lateral border of the male square buttocks. This depression is key for preserving a masculine look because it is more pronounced in men than in women.^4^

The lateral gluteal depression, although considered the safest area for injection, proves more challenging to expand compared to the general gluteal area. This zone is constituted by the top two‐thirds of the buttocks, positioned laterally to the hazard zone. Remarkably, it lacks significant ligamentous or fibrous attachments, embodying only variations in superficial dermal and subcutaneous density.^22^ As such, the authors propose two latero‐medial entry points to facilitate easier accessibility. For this specific technique, to avoid the muscle aponeuroses' orientation, the authors selected strategic entry points. This selection notably facilitates application and enables the crossing of planes to achieve more uniform results. The authors opted for the use of 18/70 cannulas. As per research, the use of a cannula ensures increased safety, aids in reducing haematomas and expedites recovery.^23^

Various buttock enhancement techniques have been performed over the years. As stated in the United States cosmetic surgery statistics, implant‐based gluteoplasty and autologous fat grafting are the most frequently employed techniques.^24^ However, gluteal augmentation with implants is associated with an overall complication rate of 30.5%.

Additionally, fat grafting is commonly used to fill the gluteal area.^25^ Oranges et al.^24^ reported an overall complication rate of 10.5% in patients undergoing autologous fat grafting. Moreover, autologous fat grafting requires liposuction, unlike HA injection, making it a viable procedure for slim patients without a donor area.

Injectable fillers, based on HA, demonstrate many properties of an ideal filler: they possess a simple and reproducible technique, are made from a biocompatible material, are non‐toxic, and can be easily removed if necessary.^9^

In recent years, new forms of HA have been developed. The author described a new technique for buttock augmentation using a large and stable molecule of HA gel with a high G prime in a previous article. This technique involves using 20 mL of HA per session for the entire gluteal region. If necessary, the authors recommend scheduling a new session only after 15–30 days, as this product has a 30% volumetric restoration factor,^9^, ^13^ thereby increasing the final volume by an additional 30% in the days following the procedure. The injection was planned to be subcutaneous where no major blood vessels were located.^26^

The HA employed has large particles, with 70% comprising 1800 micromeres and 30% ranging from 800 to 1800 micromeres. The slight discrepancy in particle size enriches the product's viscoelastic properties, enabling superior tissue adaptation despite the particles' substantial size. Previous ultrasound studies evidenced that this product consistently flows through the cannula, distributing both downwards and upwards uniformly, in a pseudocystic string manner.^27^ This characteristic allows the subcutaneous use of the product without inducing surface irregularities on the skin.

Another significant feature of the utilized product is its large particle composition. Each particle boasts a firm centre encased by a softer periphery. This characteristic paves the way for exceptional tissue adaptation, enabling subcutaneous use without evident visibility or palpability. Further, the sturdy centre of these particles in the product affords durable outcomes, observable after an initial 10‐month follow‐up.^28^ Previous studies on other large particle HA revealed substantial satisfaction rates, extending up to 24 months.^9^, ^12^ In the lateral depression area, the lack of subcutaneous tissue is common, presenting a significant issue for the tissue's adaptive capacity.

The importance of emphasizing that HA is a non‐autologous and resorbable product cannot be overstated; therefore, the technique must be performed regularly to maintain the results.^9^, ^12^, ^26^

In recent years, HA has been utilized in body treatments.^27^, ^28^ The authors propose that additional studies be conducted in other body areas, considering its unique, intriguing and promising rheology.^9^, ^12^, ^26^

## 5 CONCLUSION

The hip dips technique, which utilizes HA with large‐size particles to augment lateral depression, has been demonstrated to be a minimally invasive technique offering rapid results with minimal risks and downtime. It has resulted in patient satisfaction by enhancing their self‐esteem and quality of life.

## CONFLICT OF INTEREST STATEMENT

The authors declare no conflicts of interest.

## AUTHOR CONTRIBUTIONS

**Luciana Lourenco**: Conceptualization (equal); data curation (equal); formal analysis (equal); funding acquisition (equal); investigation (equal); methodology (equal); project administration (equal); resources (equal); software (equal); supervision (equal); validation (equal); visualization (equal); writing—original draft (equal); writing—review and editing (equal). **Helena Medeiros**: Conceptualization (equal); investigation (equal); project administration (equal); resources (equal); software (equal); writing—review and editing (equal). **Luísa Ferreira**: Conceptualization (equal); investigation (equal); resources (equal); software (equal); writing—review and editing (equal). **Natasha Favoretto Dias de Oliveira**: Conceptualization (equal); formal analysis (equal); funding acquisition (equal); investigation (equal); project administration (equal); resources (equal); validation (equal); writing—original draft (equal); writing—review and editing (equal). **Roberta Lopes**: Conceptualization (equal); investigation (equal); resources (equal); software (equal); visualization (equal); writing—review and editing (equal). **Rosa Sigrist**: Data curation (equal); formal analysis (equal); investigation (equal); project administration (equal); validation (equal); writing—review and editing (equal).

## ETHICS STATEMENT

All selected individuals read the informed consent form and agreed to participate. The study was performed in accordance with the Helsinki Declaration of 1975, as revised in 1983.

## PATIENT CONSENT STATEMENT

Written patient consent for publication was obtained.

## Data Availability

The data that support the findings of this study are openly available online.
